# Ecologic Study of Meningococcal B Vaccine and *Neisseria gonorrhoeae* Infection, Norway

**DOI:** 10.3201/eid2206.151093

**Published:** 2016-06

**Authors:** Jane Whelan, Hilde Kløvstad, Inger Lise Haugen, Mirna Robert-Du Ry van Beest Holle, Jann Storsaeter

**Affiliations:** Novartis Pharma BV,^1^ Amsterdam, the Netherlands (J. Whelan, M. Robert-Du Ry van Beest Holle);; Norwegian Institute of Public Health, Oslo, Norway (H. Kølvstad, I.L. Haugen, J. Storsaeter)

**Keywords:** Gonorrhea, incidence, Norway, epidemiology, meningococcal vaccines, *Neisseria meningitidis* serogroup B, *Neisseria gonorrhoeae*, sexually transmitted infections, bacteria, gram-negative bacterial infections

**To the Editor:** Gonorrhea is a sexually transmitted disease that can cause pelvic inflammatory disease, ectopic pregnancy, and salpingitis in women and infertility in men and women. Rates vary; incidence is 12.5 cases/100,000 population in Europe (*1*) and ≈6,000 cases/100,000 population in parts of sub-Saharan Africa (*2*). Recurrent infection is common, antimicrobial drug resistance is growing, and no licensed vaccine is available to protect against gonorrhea infection. Components of some meningococcal B (MenB) vaccines could provide protection against the causative bacterium, *Neisseria gonorrhoeae* (M. Pizza, pers. comm.), because the meningococcus bacterium is of the same *Neisseria* genus and the 2 bacteria share key protein antigens, such as the outer membrane vesicle (OMV). Ecologic evidence from Cuba supports a decline in gonococcus infection after a nationwide OMV vaccine campaign in the 1980s (*3*). In Norway, a trial of another OMV MenB vaccine (MenBvac, Norwegian Institute of Public Health, Oslo, Norway) was conducted among teenagers during 1988–1992. We retrospectively examined associations between MenB vaccine coverage during 1988–1992 and national gonorrhea rates for persons >16 years of age during 1993–2008 in Norway. 

Age, year of birth, and year of diagnosis for anonymized, laboratory-confirmed gonorrhea patients were available from the Norwegian Surveillance System for Communicable Diseases from 1993 onwards. We collected aggregate vaccination coverage by MenB vaccine from the electronic trial register for 1988–1992. Only children enrolled in secondary schools and ≈13–15 years of age were offered the vaccine. This group accounted for 63% of the 148,589 children resident in Norway during the trial period and born during 1973–1976. Using annual population estimates, we derived gonorrhea notification rates and compared 3 cohorts: the vaccinated cohort (VC) born during 1973–1976; the unvaccinated cohort born during 1965–1972 (pre-VC); and the unvaccinated cohort born after 1976 (post-VC). Using Poisson log-linear regression adjusted for year of diagnosis, we calculated incidence rate ratios (IRRs; number of new diagnoses of gonorrhea per 100,000 population) for VC and post-VC and compared them with IRRs for pre-VC (the reference group).

During 1993–2008, a total of 2,601 cases of gonorrhea were reported. To avoid case ascertainment bias, cases reported from 2009 onwards were excluded because >50% were diagnosed by PCR. In men, notification rates fell from 27.3/100,000 population in 1993 to 12.3 in 1995 but increased to 22.9 in 2008 (IRR 1.02, 95% CI 1.00–1.03, p = 0.001). The recent increase is largely attributable to transmission between men who have sex with men, a group accounting for 12% of cases among men in 1993 but 40% in 2008. Among women, incidence rates dropped from 20.7/100,000 population in 1993 to 3.1 in 1999 and have remained stable since 2008, when the rate was 4.1. 

We hypothesized that an immunogenic effect of MenB vaccination on gonorrhea notification rates might be most expected among persons in their early 20s in the mid-1990s, a group at risk for gonorrhea infection during years closest to the vaccination campaign. A small but significant reduction occurred in crude incidence rates for women 20–24 years of age in the VC (IRR 0.58, 95% CI 0.42–0.8); however, this reduction was not significant after incidence rates were adjusted for year of diagnosis (IRR 0.72, 95% CI 0.51–1.02). No change in incidence occurred for other age groups or birth cohorts among women (Figure). Reduced incidence also occurred for men 20–24 years of age in the VC (adjusted IRR 0.68, 95% CI 0.51–0.93) and persisted in the post-VC (adjusted IRR 0.51, 95% CI 0.33–0.78); the IRR increased for men 25–29 years of age in the post-VC (adjusted IRR 1.6, 95% CI 1.06-2.43). (Figure).

**Figure:**
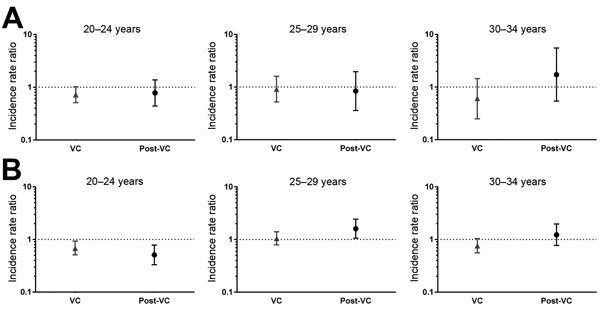
Incidence rate ratios (IRRs) of cases of gonorrhea by age group and birth cohort, adjusted for year of diagnosis in A) women and B) men, Norway. IRRs were calculated by using Poisson log-linear regression and adjusted for year of diagnosis. IRRs indicate incidence of gonorrhea in the vaccinated cohort (VC), (i.e., persons born during 1973–1976, 63% of whom were vaccinated), represented by triangles, and in the post-VC (i.e., those born after 1976), represented by circles, relative to the reference birth cohort, the pre-VC (i.e., persons born during 1965–1972), represented by the horizontal dotted line. Also shown are 95%s CI. Differences in the IRR were considered statistically significant if the CI did not include 1. A) Among women, the adjusted IRR in the VC 20–24 years was of borderline significance: 0.72, 95% CI 0.51–1.02. No effect was seen in other age groups or birth cohorts. B) Among men, a reduced adjusted incidence was evident for those 20–24 years of age in the VC (IRR 0.68, 95% CI 0.51–0.93) and persisted among post-VC who were 20–24 years of age (IRR 0.51, 95% CI 0.33–0.78). IRR increased for men 25–29 years of age in the post-VC (IRR 1.6, 95% CI 1.06-2.43).

Overall, rates of gonorrhea dropped among men and women after the vaccination campaign; however, rates had already been in decline since the mid-1970s (*4*). A limited age-specific vaccine effect occurred among men and women 20–24 years of age. No vaccine effect was found among women in other age groups or birth cohorts. Among men 20–24 years of age, the persistent decline occurring among the post-VC could be explained by a herd effect, but rates subsequently increased for men 25–29 years of age. Different effects for men and women may be explained in part by changing transmission patterns and sexual behavior occurring among men who have sex with men and by inadequate adjustment of the underlying gonorrhea trend in men; however, differences by sex are difficult to interpret. 

Among study limitations, our ecologic study design could not distinguish between long-term trends or behavioral factors and vaccine effects. For example, in the early 1990s, condom use increased in Norway, especially among persons in their early 20s (*5*), possibly in response to the evolving HIV epidemic (*6**,**7*). In addition, moderate population vaccine coverage in the VC (i.e., 63%) and a time lag between the vaccination program (1988–1992) and the start of surveillance (1993) may have diluted tangible vaccine effects. For further examination of cross-immunity of MenB vaccines with gonococci, vaccine effectiveness studies in regions where OMV vaccine was used (e.g. New Zealand) and evaluation of new protein-based vaccines are warranted. 
